# Digital Technologies for Health Promotion and Disease Prevention in Older People: Protocol for a Scoping Review

**DOI:** 10.2196/37729

**Published:** 2022-07-21

**Authors:** Karina Karolina De Santis, Lea Mergenthal, Lara Christianson, Hajo Zeeb

**Affiliations:** 1 Department of Prevention and Evaluation Leibniz Institute for Prevention Research and Epidemiology-BIPS Bremen Germany; 2 Leibniz-Science Campus Digital Public Health Bremen Bremen Germany; 3 Department of Administration Leibniz Institute for Prevention Research and Epidemiology-BIPS Bremen Germany; 4 Faculty 11 Human and Health Sciences University of Bremen Bremen Germany

**Keywords:** digital technology, health technology, digital public health, health promotion, disease prevention, healthy aging, elderly population, older adult, older population, scoping review

## Abstract

**Background:**

Digital technologies could contribute to health promotion and disease prevention. It is unclear if and how such digital technologies address the health needs of older people in nonclinical settings (ie, daily life).

**Objective:**

This study aims to identify digital technologies for health promotion and disease prevention that target the needs of older people in nonclinical settings by performing a scoping review of the published literature. The scoping review is guided by the framework of Arksey and O’Malley.

**Methods:**

Our scoping review follows the PRISMA-ScR (Preferred Reporting Items for Systematic Reviews and Meta-Analyses Extension for Scoping Reviews) guidelines. The information sources are bibliographic databases (MEDLINE, PsycINFO, CINAHL, and SCOPUS) and bibliographies of any included systematic reviews. Manual searches for additional studies will be performed in Google Scholar and most relevant journals. The electronic search strategy was developed in collaboration with a librarian who performed the search for studies on digital technologies for health promotion and disease prevention targeting the needs of older people. Study selection and data coding will be performed independently by 2 authors. Consensus will be reached by discussion. Eligibility is based on the PCC (Population, Concept, and Context) criteria as follows: (1) older people (population); (2) any digital (health) technology, such as websites, smartphone apps, or wearables (concept); and (3) health promotion and disease prevention in nonclinical (daily life, home, or community) settings (context). Primary studies with any design or reviews with a systematic methodology published in peer-reviewed academic journals will be included. Data items will address study designs, PCC criteria, benefits or barriers related to digital technology use by older people, and evidence gaps. Data will be synthesized using descriptive statistics or narratively described by identifying common themes. Quality appraisal will be performed for any included systematic reviews, using a validated instrument for this study type (A Measurement Tool to Assess Systematic Reviews, version 2 [AMSTAR2]).

**Results:**

Following preliminary literature searches to test and calibrate the search syntax, the electronic literature search was performed in March 2022 and manual searches were completed in June 2022. Study selection based on titles and abstracts was completed in July 2022, and the full-text screen was initiated in July 2022.

**Conclusions:**

Our scoping review will identify the types of digital technologies, health targets in the context of health promotion and disease prevention, and health benefits or barriers associated with the use of such technologies for older people in nonclinical settings. This knowledge could guide further research on how digital technologies can support healthy aging.

**International Registered Report Identifier (IRRID):**

PRR1-10.2196/37729

## Introduction

Digital technologies, such as wearable devices, smartphone apps, and health websites, could contribute to health promotion and disease prevention in the general population [1]. In particular, younger, more educated, and wealthier members of the general population use digital technologies for healthy lifestyle promotion and report higher perceived digital health literacy [2]. Due to aging of the world population, digital technologies for healthy lifestyle promotion should also target the specific needs of older people. However, it is unclear if and what digital technologies exist for this population.

In the digitized world, surprisingly, little is known about the needs of older people regarding their use of digital technologies for healthy lifestyle promotion [3]. Although older people are considered “nondigital natives” and their use of digital technologies is associated with various barriers, such technologies could also facilitate healthy aging via access to health information and the provision of health care [4,5]. A research focus on this population is important to better understand how older people use and engage with digital technologies for healthy aging [6]. For example, access to digital health offers is possible only if older people possess appropriate technological devices, such as tablets or smartphones [7,8]. The initial adoption of such technologies depends on their acceptance by the target population. Digital technologies for older people should be easy to learn and explicitly communicate their usefulness to users [9]. Finally, sustained engagement with the technologies is necessary for their successful use. For example, adequate digital health literacy [10] and human support [11,12] are required to operate and potentially benefit from digital technologies. In general, co-creation and feedback from older users are required to develop appropriate digital technologies for healthy aging in this target population [13-15]. Furthermore, evaluation of cost-effectiveness [16] and user outcomes in the context of health promotion and disease prevention [17] is required to better understand if and how digital technologies work.

Recent scoping reviews suggest that digital health technologies for older people are used predominantly in the clinical context of disease management. For example, digital technologies may elicit behavioral changes across a range of health conditions that are required to improve disease and medication management among people aged 60 years or above [18]. Furthermore, interventions for health promotion and disease prevention targeting the needs of older people are often nondigital (only 12 out of 486 reviews addressed eHealth interventions for this population) [19]. Finally, the most common health target of digital interventions for health promotion and disease prevention is physical activity, according to a scoping review [20] and recent systematic reviews that assessed the efficacy of such interventions in older people (Table 1). As illustrated in Table 1 and discussed in other scoping reviews [17,21], the terminology in the field of digital health promotion and disease prevention is highly diverse, and a uniform definition of the age of older people does not exist. Therefore, a new scoping review is required to more broadly identify any available digital technologies that target any aspects of healthy aging in nonclinical settings (ie, daily life).

**Table 1:** Selected systematic reviews on digital technologies for older people.

Review citation	Population age (older people; years)	Digital technologies	Health outcomes
Muellmann et al, 2018 [22]	55+	eHealth	Physical activity
Buyl et al, 2020 [23]	50+	eHealth	Physical activity, diet, quality of life, and well-being
Kwan et al, 2020 [24]	50+	eHealth	Physical activity
McGarrigle et al, 2020 [25]	50+	mHealth^a^ and eHealth	Physical activity
Stara et al, 2020 [26]	50+	Digital coaching	Physical activity, healthy eating, stress management, and tobacco cessation
Janhunen et al, 2021 [27]	60+	Exergaming	Walking
Nunez de Arenas-Arroyo et al, 2021 [28]	55+	eHealth	Physical activity

^a^mHealth: mobile health.

This study aims to identify digital technologies for health promotion and disease prevention that address the needs of older people using a scoping review of published literature. The scoping review is guided by a framework for scoping studies proposed by Arksey and O’Malley [29].

Our broad objectives are to identify and examine the research activity in the field of digital technologies for health promotion and disease prevention that target the needs of older people in nonclinical settings and to identify evidence gaps that could guide future research. The scoping review will address the following specific objectives: (1) to identify the existing digital technology types (eg, smartphone apps, websites, and wearables) for health promotion and disease prevention that target the needs of older people, (2) to describe the health context of such digital technologies, including health targets (eg, physical activity, nutrition, and cognition) and health purposes (eg, mobility promotion and lifestyle monitoring), (3) to describe the target populations of such technologies in terms of sociodemographic characteristics (especially age), health status (ie, healthy, at risk for any disease, or with any disease), and settings with nonclinical focus (eg, daily life, home, or community), (4) to assess the use pattern in terms of any health benefits or barriers associated with the use of such technologies for older people in nonclinical settings, and (5) to identify any evidence gaps.

## Methods

### Study Design

Our scoping review follows the PRISMA-ScR (Preferred Reporting Items for Systematic Reviews and Meta-Analyses Extension for Scoping Reviews) guidelines [30]. The PRISMA-ScR checklist will be reported in an appendix.

### Protocol and Registration

This protocol was written before the study commenced (ie, before the electronic literature search was performed). The study was registered at the Open Science Framework [31].

### Eligibility Criteria

The eligibility for our scoping review is based on the PCC (Population, Concept, and Context) criteria (Textbox 1).

Detailed definitions of the PCC criteria are provided in Textbox 2.

**Eligibility criteria for the scoping review.**

**Inclusion criteria**

1. Population: older people
2. Concept: digital health technologies
3. Context: health promotion and disease prevention
4. Setting: nonclinical (eg, daily life, home, and community)
5. Study type: primary studies with any design or data type (quantitative and qualitative) and reviews with systematic methodology
6. Publication status: published in a peer-reviewed journal
7. Publication language: English, German, or French
8. Full-text accessible

**Exclusion criteria**

1. Older people not included
2. Digital health technologies not included
3. Other context than health promotion and disease prevention
4. Clinical setting (eg, aged care and clinical facility)
5. Other study types: protocols or narrative reviews
6. Other publication status: published without peer review, dissertations, books, conference papers, comments, corrections, letters, and editorials
7. Publication language other than English, German, or French
8. Full-text not accessible

**Definitions of the inclusion criteria in the scoping review.**

**Population: older people**

- Older people include populations with the age range defined by study authors.
- Studies with older people of any gender or health status (ie, healthy, at risk for any disease, or with any disease) will be included.
- Studies will be excluded if older people are not the focus of the study (eg, focus on carers of older people) or if they are included among people of any age or with middle-aged adults.

**Concept: digital health technologies**

- Digital technologies are defined as any digitally supported health technologies. These technologies may include the components of (1) eHealth, that is, the use of information and communications technology to support health and (2) mobile health (mHealth), that is, the use of digital devices or tools with mobile and wireless technologies to support health objectives according to a World Health Organization guideline on digital interventions [32].
- Studies will be included if they use any “traditional” technologies, such as websites accessed via computer or mobile telephones, or any “modern” technologies, such as smartphone apps, wearables, or exergaming.
- Studies will be included if digital health technologies are used alone or as part of a health intervention.
- Studies will be excluded if landline telephones are used as the main technology in the health context.

**Context: health promotion and disease prevention**

- Health promotion and disease prevention (primary, secondary, or tertiary) will be defined as any measures used to improve or maintain healthy lifestyle, prevent the onset of new diseases, or prevent worsening of existing diseases.
- Studies will be included if they focus on different aspects of healthy aging, such as physical activity, nutrition, mental and cognitive functioning, or sleep.
- Studies will be excluded if they focus on dental health, disease management, or digital technology development.

Since our scoping review focuses on 3 broad topics (digital technologies, health promotion and disease prevention, and older people), we aim to identify primary studies with any design (randomized or nonrandomized with quantitative or qualitative data) and reviews with a systematic methodology (rapid, scoping, systematic, or overview of reviews). This approach will assure that the relevant literature will be identified either in our literature search or in other reviews. Furthermore, identification of other reviews in this rapidly developing field can potentially reduce research waste that occurs when new reviews are produced, although reviews on similar topics already exist [17,33].

### Information Sources

The information sources for our scoping review are (1) 4 international bibliographic databases (MEDLINE through OVID, PsycINFO through OVID, CINAHL through EBSCO, and SCOPUS), (2) bibliographies of any included systematic reviews, (3) Google Scholar, and (4) most relevant journals in the field. These databases were chosen because they identified the most relevant literature in our searches for digital technologies in other public health contexts. Due to potential financial interests in the field of digital health technologies, grey (nonpeer reviewed) literature will not be searched for. Instead, we aim to locate only published and peer-reviewed literature that may critically and objectively evaluate the health applications of such technologies in older people.

### Search Strategy

The electronic search strategy was developed in collaboration with an experienced librarian on our team who also performed the search and deduplicated the results. The development and reporting of the search strategy adheres to PRESS (Peer Review of Electronic Search Strategies) [34] and PRISMA-S (PRISMA Statement for Reporting Literature Searches in Systematic Reviews) [35] guidelines. The preliminary search syntax was developed based on our own syntax from a scoping review on the evaluation of digital interventions for physical activity promotion [17,36] and the search strategies reported in other relevant reviews (Table 1). According to the PRESS guideline [34], we first adapted the preliminary syntax to match our PCC criteria (Textbox 1) as follows: (1) digital technologies, (2) older people, and (3) health promotion and disease prevention. Preliminary literature searches were performed in MEDLINE throughout January to February 2022 to derive the most optimal combination of search terms and calibrate the search syntax. The searches were performed by the librarian, and the results were imported into EndNote X9 (Clarivate) and sorted by publication date. The oldest and newest sources were screened for relevance by 1 author, and feedback was given to the librarian. Once a fortnight, the search syntax and the relevance of results were discussed in the team. Following our discussion, the search syntax was adjusted and tested by the librarian in MEDLINE. The impact of syntax changes on the results was assessed by 1 author and subsequently discussed in the team.

The following aspects of the search syntax were implemented according to the PRESS guideline [34] and verified during our team discussions: (1) quality of translation of the research question into search terms done by inspecting the number of hits per syntax line, (2) appropriate use of adjacency proximity operators done by comparing the number of hits following different adjacency limits, (3) choice of subject headings done by inspecting the number of hits per syntax line, (4) text word searching done by inspecting the truncation and inclusion of British and American spellings, and (5) spelling and any syntax errors done by reading the syntax strategy line by line and inspecting the use of Boolean operators and brackets. There were no filters or limits used in the syntax.

Once consensus on the most effective search strategy was reached and approved by the team, the search strategy from MEDLINE was adapted to each other database individually by the librarian. A summary of the search strategy is reported in Table 2, and the full search strategy will be reported in an appendix.

**Table 2:** Summary of the search strategy in our scoping review.

Variable	Search topic 1: Digital technologies	Search topic 2: Older people	Search topic 3: Health promotion and disease prevention
Example search terms	Telemedicine, mobile applications, internet-based or digital intervention, fitness trackers, wearables, video games, or social media	Older, elderly or senior separated by up to three terms from people, adults, or population	Health (promotion or prevention)
Search fields	Titles or abstracts	Titles or abstracts	Titles, abstracts, or keywords
Comments	Relevant MeSH (Medical Subject Headings) terms were selected in MEDLINE and corresponding subject headings were selected in PsycINFO and CINAHL.	Adjacency with up to three terms was used to identify sources in which the words “older people” were separated by additional terms, such as older “healthy” people.	We relied on author keywords or database classification systems to identify sources investigating health promotion and disease prevention, even if these terms were not used in titles or abstracts.

The electronic literature search was performed in each database separately from database inception through March 3, 2022. The search results were exported into individual libraries in EndNote and subsequently merged into a single library. This single library was exported into deduplication software Deduplicator [37], automatically deduplicated, and manually checked, and following duplicate removal, the results were imported back into a new library in EndNote.

### Selection of Sources of Evidence

Study selection will be performed in EndNote by 2 authors independently, and final consensus will be reached by discussion. The study selection procedure will involve automatic and manual elements as follows. First, all sources from the electronic search will be automatically divided into 2 libraries using the smart groups function in EndNote by 1 author as follows: (1) library I1 with sources fulfilling the inclusion criterion 1 (with the words “older people” in titles; Textbox 1) and (2) library E1 with sources fulfilling the exclusion criterion 1 (without the words “older people” in titles; Textbox 1). All titles in library E1 will be manually screened for inclusion by 2 authors independently. Sources will be excluded if other populations, such as adolescents, are mentioned in titles. Abstracts will be read if the titles do not mention the study population. Any sources that focus on older people in library E1 will be moved to library I1 for further inspection. Library I1 will be subdivided into further libraries using the smart groups function in EndNote by 1 author. For example, a smart group of sources with the term “protocol” in titles or abstracts will be created within library I1 and exported into a new library E5. Titles of sources in library E5 will be manually screened by 2 authors independently to confirm that sources identified by EndNote as study protocols were indeed study protocols. Any incorrectly classified sources will be moved back to library I1 for further inspection. This procedure will continue until all sources in library I1 will be either selected for full-text inspection (located in library I1) or excluded based on title or abstract screening and moved to libraries E1 to E7.

Following the title and abstract screening, full-text inspection of all sources in library I1 will be done manually by 2 authors independently. Consensus will be reached by discussion.

Once study selection from the database searches is complete, supplementary searches for additional studies will be performed by 1 author, and another author will check and approve the selection. Manual searches will be performed using bibliographies of any included systematic reviews. Additional searches will also be performed using Google Scholar and the websites of the most relevant journals in the field of digital public health identified in another scoping review [38] or suggested by the peer reviewers of this article. The following journals will be searched: JMIR mHealth and uHealth, Journal of Medical Internet Research, BMC Public Health, JMIR Aging, The Lancet Digital Health, PLOS Digital Health, and Frontiers in Digital Health.

A summary of study selection will be reported on the PRISMA (Preferred Reporting Items for Systematic Reviews and Meta-Analyses) flowchart. A list of included and excluded studies and reasons for exclusion after the full-text assessment will be reported in an appendix. Study selection from the electronic search was initiated in March 2022.

### Data Charting

Data coding will be performed using a single spreadsheet (Excel, version 10; Microsoft Corp) that will be developed and calibrated within the team. If necessary, a coding manual will be developed to assure high interrater reliability of coding. Data coding will be performed by 2 authors independently, and consensus will be reached by discussion.

### Data Items

A list of data items (Textbox 3) will be developed by 2 authors to address the objectives of our scoping review. If applicable, data will be coded quantitatively into predefined categories or qualitatively using author statements. Data items addressing the overlap in primary studies will be used to assess the uniqueness of evidence among the included reviews and between our electronic search and the included reviews. Specifically, the primary studies included in each review will be inserted into an additional spreadsheet (Excel, version 10) and manually compared among the reviews (sorted from the oldest to the newest) and against our list of included studies. Any primary studies included in only 1 review will be classified as unique. We will also assess the overlap in primary studies between our electronic search and the included reviews. Any primary studies identified in our search but not included in any review will be classified as unique. All coded data will be reported in an appendix.

**Data items in the scoping review.**

**Bibliographic information**

- First author, publication year, publication date, corresponding author region, title, and funding sources

**Study design**

- Study type: primary study or review
- Primary study design: randomized or nonrandomized
- Primary study data type: quantitative, qualitative, or mixed
- Review type: rapid, scoping, systematic, or overview of reviews
- Primary studies in reviews: number per review, overlap in primary studies among all reviews, and overlap in primary studies among reviews and our electronic search

**Study aim and focus**

- Study aim according to authors
- Study focus: evaluation, feasibility, efficacy, or other

**Population (older people)**

- Sample size
- Sociodemographic characteristics: age, gender, and others (eg, working or retired, socioeconomic status, country of data collection, and digital health competence)
- Health status: healthy (without or at risk for any disease) or clinical (with any disease)
- Setting: daily life, home, community, or others with examples

**Concept (digital technology)**

- Type: any digital technology (telemedicine, eHealth, or mHealth), wearable device, smartphone or other mobile tool, app, internet, website, exergaming, virtual reality, or others with examples

**Context (health promotion and disease prevention)**

- Health target: physical activity, nutrition, mental and cognitive functioning, sleep, or others with examples
- Health purpose: healthy lifestyle promotion, disease prevention (primary, secondary, or tertiary), lifestyle monitoring, reminders, performance feedback, social or virtual network development, or other

**Use pattern (benefits vs barriers)**

- Duration
- Benefits (eg, acceptability, engagement, and outcome evaluation)
- Barriers (eg, reasons for attrition and difficulties with use)

**Evidence gaps**

- Study conclusions or author statements focusing on ideas for future research

### Critical Appraisal of Individual Sources of Evidence

Except for systematic reviews, the critical appraisal of included studies will not be performed because our scoping review aims to broadly identify digital technologies for older people rather than to evaluate their efficacy in the context of healthy aging. The quality of existing evidence will be discussed based on study designs identified in the scoping review.

The critical appraisal of systematic reviews will be performed according to guidelines for overviews of systematic reviews [39] with a validated tool for systematic reviews (A Measurement Tool to Assess Systematic Reviews, version 2 [AMSTAR2] [40]). AMSTAR2 consists of 16 items (7 critical and 9 noncritical). The appraisal outcome is the overall confidence rating in the results of a systematic review (critically low, low, moderate, or high) based on a combination of scores on critical and noncritical items [40]. Critically low ratings are assigned if at least two critical items are not fulfilled (rated as no) on AMSTAR2.

The appraisals will be performed according to a 2-step procedure described in our protocol for another scoping review [36]. In the first step, 2 items on AMSTAR2 (item 2: presence of a review protocol and item 7: presence of a list of excluded studies) will be rated to identify any systematic reviews with critically low confidence ratings. These 2 items were chosen because they are typically not fulfilled in systematic reviews of nondigital or digital health interventions [17,33,41]. In the second step, any systematic reviews that fulfill item 2, item 7, or both will be rated with all 16 AMSTAR2 items according to AMSTAR2 guidance [40].

A spreadsheet (Excel, version 10) will be developed and used for appraising systematic reviews with AMSTAR2. All systematic reviews will be independently appraised by 2 authors, and consensus will be reached by discussion. The overall confidence ratings for each systematic review will be reported in an appendix.

### Synthesis of Results

Data will be synthesized according to the objectives of our scoping review. The quantitative data items and AMSTAR2 appraisal outcomes for all systematic reviews will be synthesized using descriptive statistics (frequencies, means, and SDs, if applicable). The qualitative data items will be narratively described by identifying common themes.

## Results

Following preliminary literature searches to test and calibrate the search syntax, the electronic literature search was performed in March 2022 and manual searches were completed in June 2022. Study selection based on titles and abstracts was completed in July 2022, and the full-text screen was initiated in July 2022.

## Discussion

### Principal Findings

Our electronic search identified just over 2000 sources. Study selection is expected to be completed in July 2022. The smart groups function in EndNote helped us to initially manage and automatically sort the literature. EndNote was very precise at identifying certain publication types, such as reviews, study protocols, dissertations, books, and conference papers. EndNote also helped us to identify sources with other populations, such as young people, and other settings, such as aged care. Most human judgement was required to decide if technologies used in studies were digital, and if so, if they were used in the context of health promotion and disease prevention. Furthermore, the study population was not mentioned in the titles of about 25% of search results, and the abstracts of these studies had to be manually assessed. So far, there have been only few minor disagreements between the 2 authors involved in the title and abstract screening. These disagreements were resolved by discussion between both authors based on additional information for or against inclusion.

Fully automated and preliminary sorting of studies into smart groups in EndNote showed that various digital technologies are used for health promotion and disease prevention by older people, including any technologies (digital, virtual, video, eHealth, or telehealth), websites accessed via a computer, SMS (text messages) or mobile phones, exergaming, smartphones, or wearables. The studies addressed different health targets, including physical activity, mental health and wellness, nutrition, and cognitive functioning. The study focus is on effectiveness, feasibility, or evaluation of digital technologies.

### Comparison With Prior Work

Two interesting aspects of our scoping review are to identify the digital technologies preferred by older people and to assess the reasons for using such technologies in the health context. Our preliminary inspection of studies suggests that while older people may use more modern technologies, such as smartphone apps or wearables, they also use (and possibly prefer) other technological solutions and devices, such as websites accessed via computers. Furthermore, while physical activity was the primary focus of previous reviews in this field (eg, the review by Taylor et al [20] and reviews listed in Table 1), healthy aging is associated with various health outcomes. According to our preliminary inspection of studies, these may also include nutrition, and mental and cognitive functioning. It is likely that we will identify other aspects of health promotion and disease prevention in the final sample of studies, including weight management, substance use prevention, sleep monitoring, and promotion of social functioning.

### Strengths and Limitations

The main strength of our scoping review is the electronic search syntax that was iteratively tested and revised by an experienced librarian on our team. Regardless of frequent piloting, there are several potential limitations in our search strategy, meaning that we might have missed some relevant studies in this new field. First, the research field of digital health promotion and disease prevention [21] and the terminology in this field [17] are highly diverse and nonstandardized. For example, 40 reviews of similar (digital) interventions in the same field (physical activity promotion) located different published primary studies in their searches, meaning that about 80% of studies were included in only 1 of the 40 reviews [17]. Second, the age range of older people typically varies among studies. To circumvent this problem, there is no age limitation for older people in our scoping review. Instead, the age range of participants will be coded to investigate any patterns in the results. Third, the terms “health promotion” and “disease prevention” can address very different health targets and are typically not mentioned in study titles or abstracts. Our preliminary searches showed that the most relevant studies were obtained when these terms were included in keyword searches but not when they were omitted from the search syntax. Manual searches for additional studies in other reviews or in the most relevant journals may be essential in this new field. Finally, we focus only on (objective) peer-reviewed literature published in academic journals. This choice is guided by the general difficulty in assessing any financial interests associated with digital technologies that may be present in nonacademic literature.

### Dissemination Plan

We plan to publish the results of our scoping review in a peer-reviewed academic journal and also to disseminate our findings using plain-language summaries in English and German. Such summaries may facilitate the knowledge translation from our scoping review to a broader audience, including stakeholders working in the field of health promotion or the target population of older people. In general, knowledge translation is required to successfully process scientific findings before they can be applied in practice. Public health stakeholders involved in promoting physical activity among the elderly in Germany identified such short summaries as a strategy that could aid their work [42].

### Conclusions

Our scoping review will identify the types of digital technologies, health targets in the context of health promotion and disease prevention, and use benefits or barriers for older people in nonclinical settings. This knowledge could guide further research on how digital technologies can support healthy aging.

## Abbreviations

AMSTAR2: A Measurement Tool to Assess Systematic Reviews, version 2
PCC: Population, Concept, and Context
PRESS: Peer Review of Electronic Search Strategies
PRISMA-ScR: Preferred Reporting Items for Systematic Reviews and Meta-Analyses Extension for Scoping Reviews
